# Live imaging of primary ocular vasculature formation in zebrafish

**DOI:** 10.1371/journal.pone.0176456

**Published:** 2017-04-26

**Authors:** Tetsuya Hashiura, Eiji Kimura, Shizuko Fujisawa, Sayuri Oikawa, Shigenori Nonaka, Daijiro Kurosaka, Jiro Hitomi

**Affiliations:** 1 Department of Anatomy, Iwate Medical University, 2-1-1 Nishitokuta, Yahaba-cho, Shiwa, Iwate, Japan; 2 Department of Ophthalmology, Iwate Medical University, 2-1-1 Nishitokuta, Yahaba-cho, Shiwa, Iwate, Japan; 3 Laboratory for Spatiotemporal Regulations, National Institute for Basic Biology, 38 Nishigonaka, Myodaiji, Okazaki, Aichi, Japan; Medical College of Wisconsin, UNITED STATES

## Abstract

Ocular vasculature consists of the central retinal and ciliary vascular systems, which are essential to maintaining visual function. Many researchers have attempted to determine their origins and development; however, the detailed, stepwise process of ocular vasculature formation has not been established. In zebrafish, two angioblast clusters, the rostral and midbrain organizing centers, form almost all of the cranial vasculature, including the ocular vasculature, and these are from where the cerebral arterial and venous angioblast clusters, respectively, differentiate. In this study, we first determined the anatomical architecture of the primary ocular vasculature and then followed its path from the two cerebral angioblast clusters using a time-lapse analysis of living *Tg(flk1*:*EGFP)*^*k7*^ zebrafish embryos, in which the endothelial cells specifically expressed enhanced green fluorescent protein. We succeeded in capturing images of the primary ocular vasculature formation and were able to determine the origin of each ocular vessel. In zebrafish, the hyaloid and ciliary arterial systems first organized independently, and then anastomosed via the inner optic circle on the surface of the lens by the lateral transfer of the optic vein. Finally, the choroidal vascular plexus formed around the eyeball to complete the primary ocular vasculature formation. To our knowledge, this study is the first to report successful capture of circular integration of the optic artery and vein, lateral transfer of the optic vein to integrate the hyaloidal and superficial ocular vasculatures, and formation of the choroidal vascular plexus. Furthermore, this new morphological information enables us to assess the entire process of the primary ocular vasculature formation, which will be useful for its precise understanding.

## Introduction

For most animals, vision is a critical sense for avoiding danger and obtaining food. The ocular vasculature is necessary to maintain this sense, and congenital and acquired diseases of the ocular vasculature cause visual dysfunction. Familial exudative vitreoretinopathy and retinopathy of prematurity are representative congenital diseases resulting in visual handicap. In the both cases, abnormal neovascularization of retinal vessels cause retinopathy [1, 2]. In addition, diabetes has dramatically increased in advanced nations, and diabetic retinopathy, which is a complication of the disease, will be the most common disease causing primary visual loss in the near future [3]. To overcome visual dysfunction induced by these diseases, we need to understand the regulatory mechanisms underlying ocular vascular morphogenesis, and, for this, a precise understanding of its formation will be necessary.

In the adult human, there are two ocular arterial systems for the retina: the central retinal and ciliary arterial systems. The former is distributed in the inner region of the retina, while the latter supplies blood only to the photoreceptor cells via the choroidal layer. The ophthalmic artery from the internal carotid artery supplies blood to both of these systems. Numerous studies of cerebral vascular formation during early ontogeny have been reported in several vertebrates, such as chickens, pigs, and human embryos [4–9]. Especially among them, Padget described the primary ocular vascular formation including regression of the hyaloid artery (HA) precisely by reconstructing the serial sections of human embryos [6, 7]. The HA was temporally formed to supply blood to the inner retina and hyaloid body, and its regression is necessary for normal retinal development. Abnormalities in this process cause cognitive retinopathy (persistent hyperplastic primary vitreous) [10]. Although Padget showed an outline of ocular vasculature formation, the classical methods used, such as the reconstruction of serial sections, have methodological limitations that prevent a complete understanding. The method can only be used to visualize the lumenized vasculature, not endothelial cells themselves, and do not allow continuous observation of primary vascular formation in the same embryo.

To understand the process of primary ocular vasculature formation, we focused on a zebrafish (*Danio rerio*) model. The zebrafish is an ideal model organism for developmental biology. Its transparent body and exo-uterine development enables continuous observation of primary vascular morphogenesis in a single embryo over time. A vascular atlas of developing zebrafish has been created using confocal microangiography, and a vessel nomenclature has been described for the comparison of the vascular anatomy of zebrafish with those of other vertebrate phyla [11]. By virtue of this atlas, screening of mutations and chemical reagents that are associated with vascular formation has been significantly promoted [12–14]. Furthermore, live imaging of vasculature formation was achieved by visualizing living transgenic zebrafish with multi-photon microscopy, in which the enhanced green fluorescent protein (EGFP) was specifically expressed in endothelial cells [15, 16]. Using this method, the development of the intersegmental [17] and cranial [18] vasculatures and the integration of vascular systems between the brain and spinal cord [19] have been demonstrated. In zebrafish, the two angioblast clusters, the rostral organizing center (ROC) and the midbrain organizing center (MOC), developed into almost all of the cranial vasculature, including the ocular vasculature [18]. The developmental process and genes associated with hyaloid and retinal vessel formation [20–23] and vascular morphogenesis of superficial ocular vessels [23, 24] in zebrafish have also been reported. However, the precise description of primary ocular vasculature formation was still insufficient and not fully summarized.

In this study, we performed a time-lapse analysis using transgenic zebrafish in which endothelial cells were specifically visualized by the expression of EGFP, to elucidate how the primary ocular vasculature were constructed from the ROC and MOC, which differentiated to the arterial and venous cerebral angioblast clusters. We succeeded in capturing images of its organization, and in determining the origin of each ocular vessel. The morphological information presented here will be useful in determining both the morphogenetic and pathogenic mechanisms underlying the process.

## Materials and methods

### Zebrafish (*Danio rerio*)

The *Tg(flk1*:*EGFP)*^*k7*^ [19] zebrafish embryos, in which EGFP is specifically expressed in endothelial cells, were used for multi-photon microscopy. The double *Tg(flt1_enh*:*EGFP)*^*k28*^ and *Tg(flk1*:*mCherry)*^*k6*^ zebrafish embryos were used for light-sheet microscopy to distinguish between the arterial and venous vessels. In the *Tg(flt1_enh*:*EGFP)*^*k28*^ embryos, EGFP was primarily expressed by the arterial vessels. In contrast, all the endothelial cells expressed mCherry in the *Tg(flk1*:*mCherry)*^*k6*^ embryos. The detail of DNA constructs for these transgenic zebrafish was described previously [25, 26]. Wild-type (WT) zebrafish were used for both *in situ* hybridization and the reconstruction study. For fluorescent imaging, pigmentation was suppressed by treating embryos with 1-phenyl-2-thiourea (PTU), or by using albino b4 mutant zebrafish [16]. Embryos were raised and the fish maintained following previously described methods [27]. This study was approved by the animal care ethical committee of Iwate Medical University (Permit Number: 28–030), and fish care and experimental procedures strictly followed the Guide for Animal Experimentation of the animal care ethical committee of Iwate Medical University. All experiment for time-lapse imaging was performed under tricaine anesthesia, and all efforts were made to minimize suffering.

### Multi-photon and light-sheet microscopy

Fluorescent imaging of the *Tg(flk1*:*EGF)*^*k7*^ zebrafish embryos were performed using a Zeiss LSM 510 META NLO, equipped with a Mai Tai (920 nm, Spectra Physics) laser. Objective lenses of W Plan-APOCHROMAT 20x/1.0 DIC (UV) VIS-IR (Zeiss) were used in this study. Reconstructions of three-dimensional images and the time-lapse analysis were conducted using ZEN software (Zeiss). For time-lapse sequences, Z stacks were collected at 18–35 min intervals. Results of time-lapse movies are presented as selected frames and in the S1–S6 Movies. Detail of the imaging process was previously described [19]. The double *Tg(flt1_enh*:*EGFP)k*^*28*^ and *Tg(flk1*:*mCherry)*^*k6*^ zebrafish embryos were observed with light-sheet microscopy (Lightsheet Z.1, Zeiss).

### Whole-mount *in situ* hybridization

Fragments of zebrafish *etsrp/etv2*, *hey2*, and *flt4* genes were cloned into a pCR-Blunt II-TOPO vector (Invitrogen) using the following primer sets: forward (5′-CAGTGGTGAAGACCTGTCCT-3′) and reverse (5′-GGCAATCTGCTGCAAAGTCC-3′) for the *etsrp/etv2* gene, forward (5′-CTTATCAGAGTTGCGTCGTCT-3′) and reverse (5′-GTTGTGGTGAAGGCTAGTGG-3′) for the *hey2* gene, and forward (5′-CACCAAAGACCAGCTTGTGA-3′) and reverse (5′- GTGACGGTATCGAGGTGCTT-3′) for the *flt4* gene, respectively. Anti-sense DIG-labeled RNA probes were then synthesized for the cloned fragments using a DIG RNA Labeling Kit (Roche). Whole-mount *in situ* hybridization was performed as previously described [28]. Stained embryos were then mounted in 100% glycerol and imaged using a stereomicroscope (SZX16, Olympus) equipped with a digital camera (DP70, Olympus).

### Three-dimensional reconstruction

Zebrafish embryos (wild-type) at 1and 2 day post fertilization (dpf) were embedded in glycol methacrylate (GMA) plastic or TECHNOVIT 7100 for serial semi-thin sections. Embryos were first fixed with 2.5% glutaraldehyde and 1% paraformaldehyde in 0.1M phosphate buffer (pH 7.4) for the GMA-embedding, or with 2.5% glutaraldehyde, 1% paraformaldehyde, and 3% sucrose in 0.06 M phosphate buffer (pH 7.4) for the TECHNOVIT 7100-embedding, at 4°C overnight. They were then post-fixed with 1% osmium tetroxide in 0.1 M phosphate buffer (pH 7.4) at 4°C for 2 h. After dehydration with an ethanol and 2-propanol series, fixed embryos were embedded in the GMA plastic or TECHNOVIT 7100 following the standard protocol. Semi-thin sections of 3-μm thickness were obtained using a rotary microtome (RM-2255, Leica). After staining with 0.05% toluidine blue, sections were imaged using a light microscope (BX51, Olympus) equipped with a digital camera (DP50, Olympus). Serial digital images were then loaded into Amira (Visage Imaging Inc.) with an appropriate voxel size to perform the three-dimensional reconstruction. The artery, vein, and optic vesicle in each image were respectively colored in red, blue, and green, and then reconstructed.

## Results

### Anatomy of the primary ocular vasculature in zebrafish

**Fig 1:**
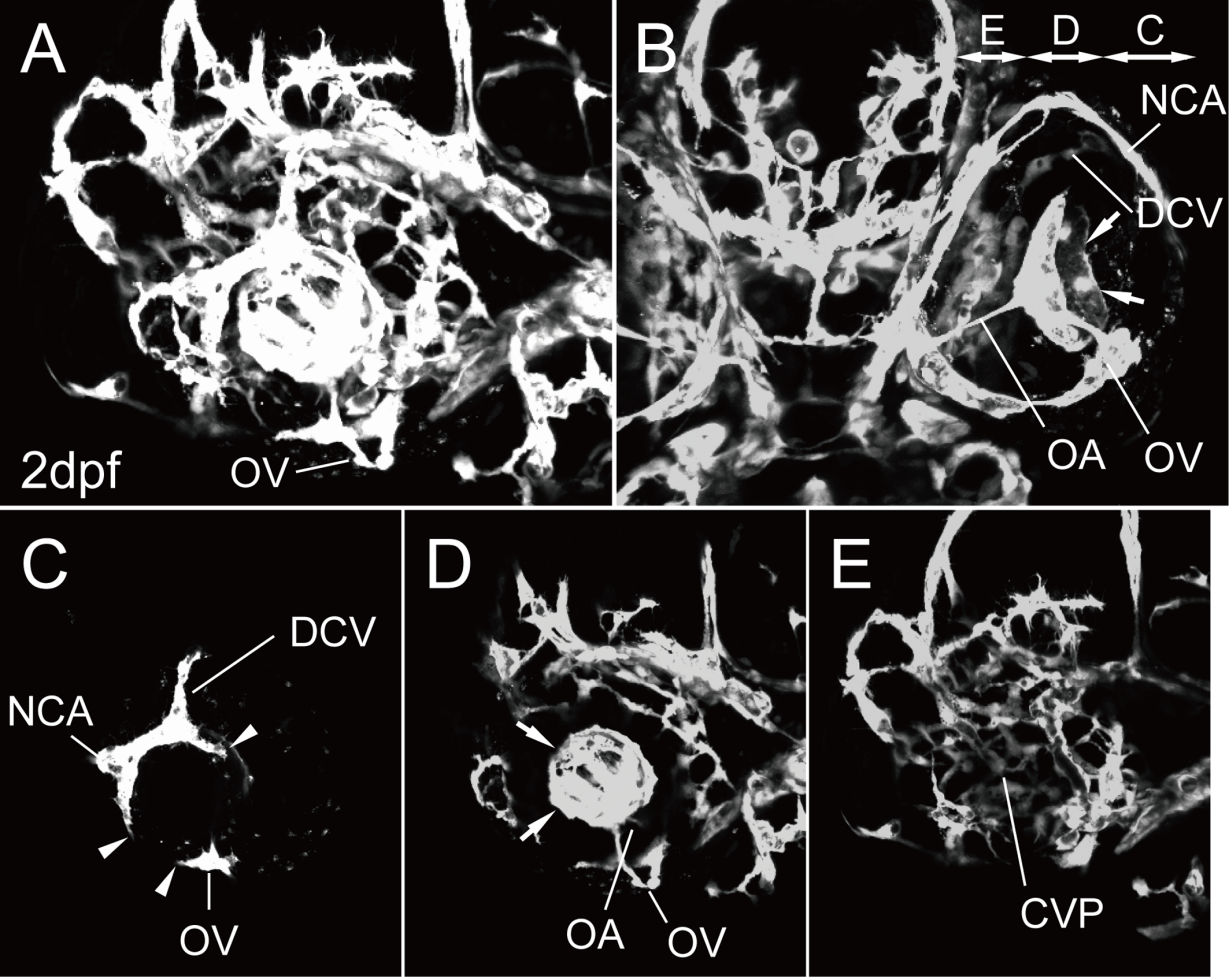
The primary ocular vasculature in zebrafish. (A–E) Ocular vascular anatomy of *Tg(flk1*:*EGFP)*^*k7*^ embryos at 2 dpf. Lateral (A, C, D, and E) and ventral (B) views. Lateral image of A divided into three parts: the superficial ocular (C) and hyaloid (D) vasculatures and the CVP (E). Panels C, D, and E in B indicate the projected region of each image. Arrows in B and D indicate the vascular plexus of the HA. Arrowheads in C indicate the IOC forming, which will connect the superficial ocular vasculature with the hyaloid vasculature.

There are two arterial systems in the ocular region of adult humans: the central retinal and ciliary arteries. The ophthalmic artery supplies blood to both systems, and the HA is temporally formed to supply blood to the hyaloidal body in early ontogeny. We first confirmed the anatomical architecture of the primary ocular vasculature in zebrafish at 2 dpf (Fig 1). There are also two vascular systems like the human: the hyaloid vascular system of the optic artery (OA) and optic vein (OV) and the ciliary vascular system of the superficial ocular vasculature and the choroidal vascular plexus (CVP) (Fig 1A–1E). The OA was connected with the OV to allow primary blood circulation to the ocular region (Fig 1D) and form the vascular plexus of the HA in the optic vesicle (Fig 1B and 1D arrows). In addition, the superficial ocular vasculature of the nasal ciliary artery (NCA) and dorsal ciliary vein (DCV) formed independently on the eyeball (Fig 1C), and these two vascular systems circularly anastomosed via the inner optic circle (IOC) at the surface of the lens (Fig 1C arrowheads). The CVP was observed around the eyeball (Fig 1E).

### Arterial and venous angioblast clusters forming the primary cerebral vasculature

**Fig 2:**
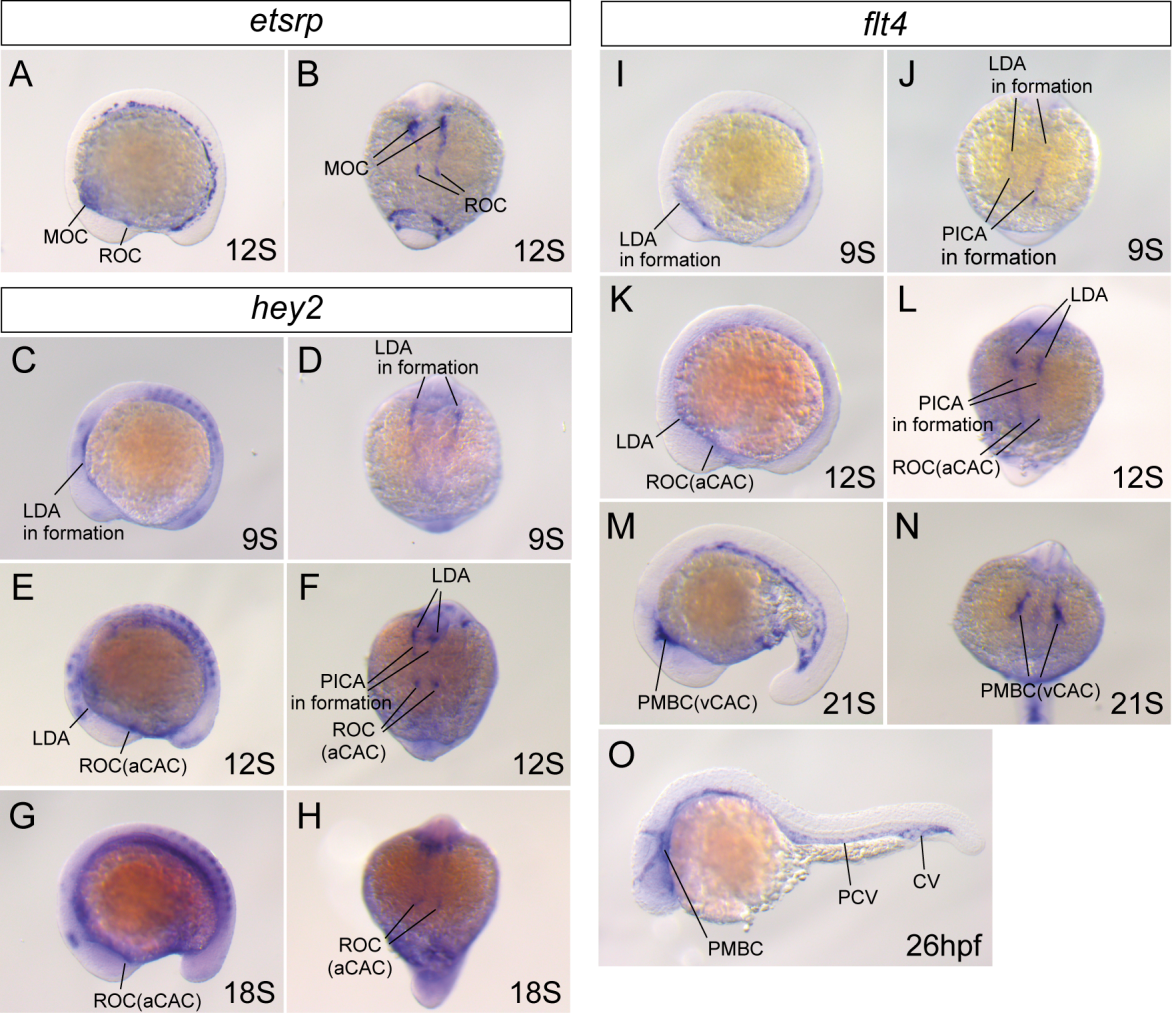
Arterial and venous cerebral angioblast clusters in zebrafish. Whole-mount *in situ* hybridization of WT embryos at 9S (C, D, I, and J), 12S (A, B, E, F, K, and L), 18S (G and H), 21S (M and N), and 26hpf (O). The expression of *etsrp/etv2* (A and B), *hey2* (C–H) and *flt4* (I–O) genes was analyzed in both lateral view (A, C, E, G, I, K, M and O) and dorsal view of the cranial region (B, D, F, H, J, L and N). The differentiation of the aCAC and vCAC from the ROC and MOC was demonstrated. PCV: posterior cardinal vein. CV: caudal vein.

The primary cerebral vasculature of the zebrafish was formed by angiogenesis from the two vascular organizing centers, the ROC and the MOC [18], which expressed the marker gene of angioblast, *etsrp/etv2* (Fig 2A and 2B). We selected the *hey2* and *flt4* genes as arterial and venous markers, respectively, and analyzed their gene expressions (Fig 2C–2O). *Hey2* gene expression was detected only in the lateral dorsal aorta (LDA) in formation at the 6-somite (S) stage (Fig 2C and 2D). As ontogeny progressed, the *hey2* gene was expressed in the LDA and primitive internal carotid artery (PICA) in formation and ROC (Fig 2E and 2F). Expression in the ROC was maintained at 18S (Fig 2G and 2H). In contrast, *flt4* gene expression was observed in the LDA and PICA in formation and ROC at 12S (Fig 2K and 2L). Their expression of the *flt4* gene gradually decreased, while strong expression of the *flt4* gene was detected in the primordial midbrain channel (PMBC) at 21S (Fig 2M and 2N), and finally detected only in the venous vessels at 26 hour post fertilization (hpf) (Fig 2O). In summary, the ROC differentiated into the arterial angioblast cluster, which we named the arterial cerebral angioblast cluster (aCAC), and a part of the MOC differentiated into the venous angioblast cluster, which we named the venous cerebral angioblast cluster (vCAC), which almost corresponded to the PMBC. To distinguish between the arterial and venous vessels of the ocular vasculature precisely, we imaged the double *Tg(flt1_enh*:*EGFP)*^*k28*^ and *Tg(flk1*:*mCherry)*^*k6*^ zebrafish embryos by light-sheet microscopy (S1 Fig). We found that the aCAC, OA, and NCA predominantly expressed EGFP, thus indicating their arterial characteristics. Conversely, the vCAC (PMBC) and DCV expressed only the mCherry protein because of their venous characteristics. In this study, we elucidated how the primary ocular vasculature was constructed from the two cerebral angioblast clusters by a time-lapse analysis of living *Tg(flk1*:*EGFP)*^*k7*^ embryos.

### Live imaging of primary ocular vasculature formation

#### Dorsal view

**Fig 3:**
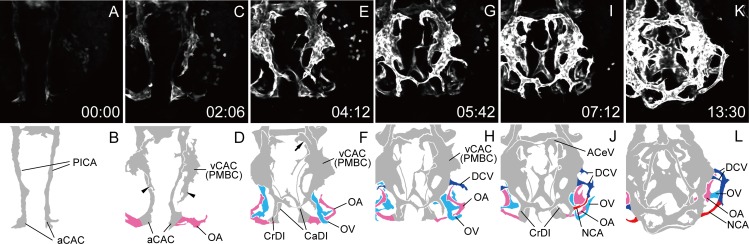
Primary ocular vasculature formation (dorsal view). Selected time-lapse images of *Tg(flk1*:*EGFP)*^*k7*^ embryo from 18S (S1 Movie) (A, C, E, G, I, and K) and their schematic diagrams (B, D, F, H, J, and L). The time (hours:minutes) from the first frame is labeled in each image (A, C, E, G, I, and K). Rostral is downward and the right side faces left. The formation of the left ocular vasculature was mainly observed. The formation of the hyaloid and ciliary vasculatures was demonstrated. Ocular vessels in the schematic diagrams are colored (NAC: red, DCV: blue, OA: pink, and OV: sky blue). Arrowheads in D indicate the rostral branch from the vCAC (PMBC), which anastomosed with the cranial division (CrDI) from the aCAC. Arrow in F indicate the medial branch from the vCAC (PMBC), which extended dorsally and anastomosed with the opposite branch at the midline to form the ACeV.

We performed time-lapse observation of primary ocular vasculature formation using a *Tg(flk1*:*EGFP)*^*k7*^ embryo in a dorsal view (S1 Movie). At 18S, only the two arterial components were observed in the cranial region, aCAC and PICA. PICA had extended rostrally and connected with aCAC on both sides (Fig 3A and 3B). *Flk1* gene expression in the vCAC (PMBC) was not detected at this stage, although undifferentiated angioblasts were present in this region, because *etsrp/etv2* gene expression was detected (Fig 2A and 2B). As ontogeny progressed, vCAC (PMBC) organization from the MOC was observed on the lateral side of the neural tube between the optic vesicle and the otocyst (Fig 3C and 3D). Two dorsal branches from the vCAC (PMBC) were observed; the rostral branch anastomosed with the cranial division (CrDI) from the aCAC (Fig 3D arrowheads), and the medial branch extended dorsally and anastomosed with the opposite branch at the midline to form the anterior cerebral vein (ACeV) (Fig 3F arrow). Arterial branches from the aCAC were also observed. The OA (pink vessels) extended to the optic vesicle, and the caudal division (CaDI) extended to the midline (Fig 3A–3F). The OV (sky blue vessels) from the ventral part of the vCAC (PMBC) also penetrated the optic vesicle and connected with the OA (Fig 3E–3J). After the primary integration of the OA and OV, the superficial ocular vasculature was then organized from the vCAC (PMBC). First, the DCV (blue vessels) sprouted from the vCAC (PMBC) laterally and reached the edges of the lens (Fig 3G and 3H). The DCV then extended rostrally and connected with the junctional point of the CrDI and vCAC (PMBC) to form the NCA (red vessels) (Fig 3I and 3J). These results indicated that both the NCA and DCV of the superficial ocular vasculature organized from the vCAC (PMBC). Although the extension of the left CrDI was delayed in this case, usually it first extended dorsally from the aCAC and anastomosed with the rostral branch from the vCAC (PMBC) as seen on the right side. The HA from the OA started to form the vascular plexus and the OV seemed to transfer to the lateral side, although ventral vascular morphogenesis could not be visualized in the simple dorsal view (Fig 3K and 3L).

**Fig 4:**
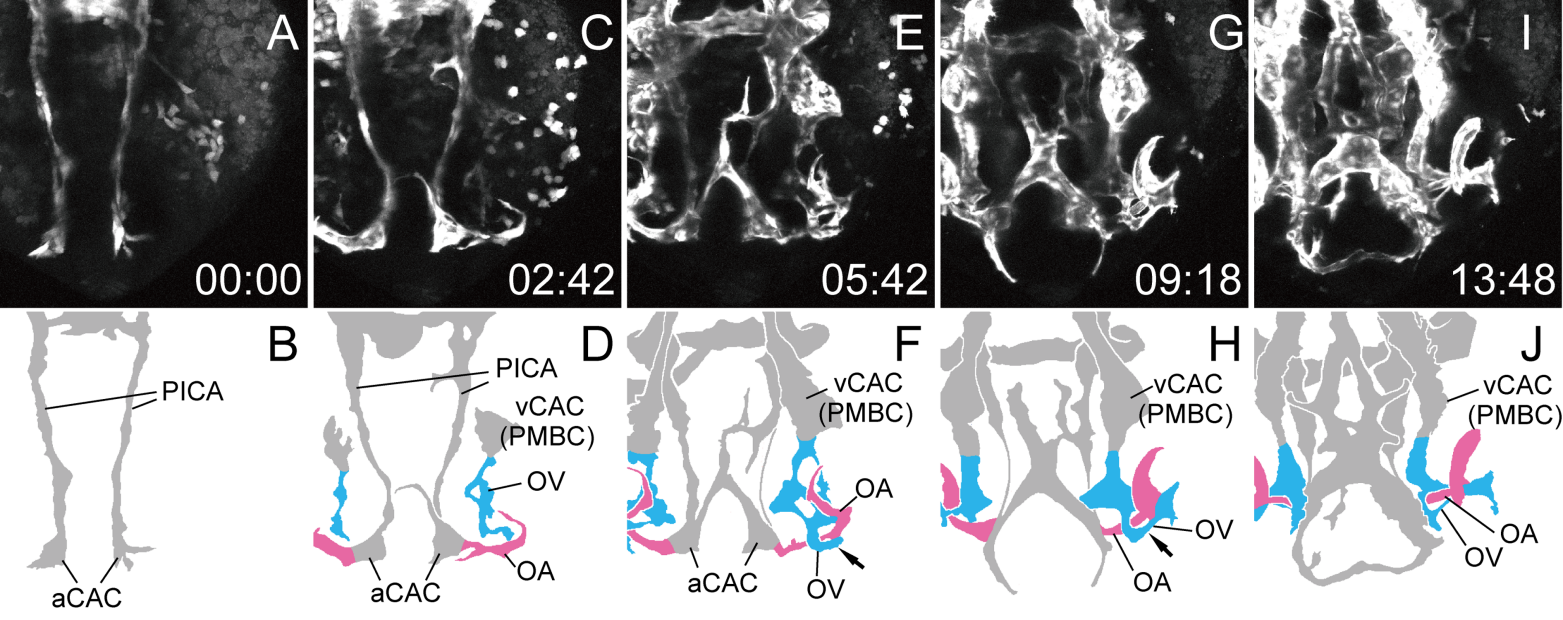
The OV from the vCAC formed the vascular plexus surrounding the OA. Selected time-lapse images of a living *Tg(flk1*:*EGFP)*^*k7*^ embryo from 18S (S2 Movie) (A, C, E, G, and I) and their schematic diagrams (B, D, F, H, and J). The time (hours:minutes) from the first frame is labeled in each image (A, C, E, G, and I). Rostral is downward and the right side faces left. The formation of the left ocular vasculature was mainly observed. To visualize ventral vascular formation, only ventral slices were extracted from S1 Movie. Ocular vessels in the schematic diagrams are colored (OA: pink, and OV: sky blue). Arrows in F and H indicate the vascular plexus of the OV surrounding the OA.

To visualize faint vessels in the ventral region, we extracted only the ventral slices from each z-stack of S1 Movie, rearranged the gain, and offset scores, and produced S2 Movie. In this movie, the extension of the OV from the ventral side of the vCAC (PMBC) (Fig 4C–4F) and the integration of the OA and OV which formed the vascular plexus around the OA were clearly demonstrated (Fig 4F and 4H arrows). The vascular plexus of the OV was finally differentiated into the CVP, as described below. The HA following the OA also formed the vascular plexus inside of the optic vesicle, and the OV transferred laterally (S2 Movie).

#### Lateral view

**Fig 5:**
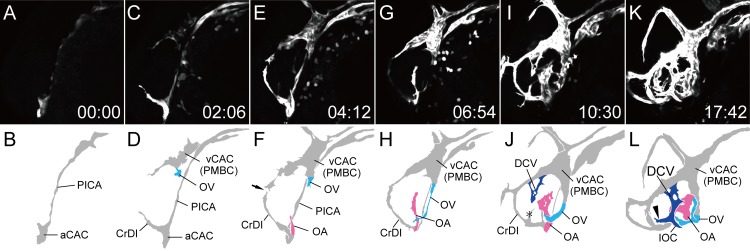
Primary ocular vascular formation (lateral view). Selected time-lapse images of a living *Tg(flk1*:*EGFP)*^*k7*^ embryo from 15S (S3 Movie) (A, C, E, G, I, and K) and their schematic diagrams (B, D, F, H, J, and L). The time (hours:minutes) from the first frame is labeled in each image (A, C, E, G, I, and K). Rostral is facing left and dorsal is facing upward. The formation of the left ocular vasculature was mainly observed. Ocular vessels in the schematic diagrams are colored (DCV: blue, OA: pink, and OV: sky blue). The OV sprouting from the vCAC (PMBC) and superficial ocular vasculature formation were observed. Arrow in F indicates the vascular connection of the CrDI and vCAC (PMBC). Arrowheads in L indicate the NCA in formation. Asterisk in J indicates the temporary anastomotic branch between the CrDI and CaDI.

We also performed time-lapse observation of primary ocular vasculature formation using the same transgenic line in a lateral view (S3 Movie). The aCAC and PICA were observed first in the lateral view (Fig 5A and 5B). The CrDI extended dorsally from the aCAC and anastomosed with the rostral branch from the vCAC (PMBC) at the rostral-dorsal edge of the optic vesicle (Fig 5F arrow). The OV also started to sprout from the rostral-ventral portion of the vCAC (PMBC) (Fig 5C–5F). The thin OV from the vCAC (PMBC) extended rostral-ventrally and penetrated the optic vesicle. Note that Fig 5G was produced by projecting the selected slices of the OV to visualize its extension clearly. The extension of the OA from the aCAC to the optic vesicle was then observed as described above (Fig 5E–5H). The DCV sprouted from the vCAC (PMBC) to the surface of the eyeball (Fig 5I and 5J) and extended to the junctional point of the CrDI and vCAC (PMBC) to form the NCA (Fig 5L arrowhead). The anastomotic branch between the CrDI and the CaDI was observed temporally (Fig 5J asterisk).

**Fig 6:**
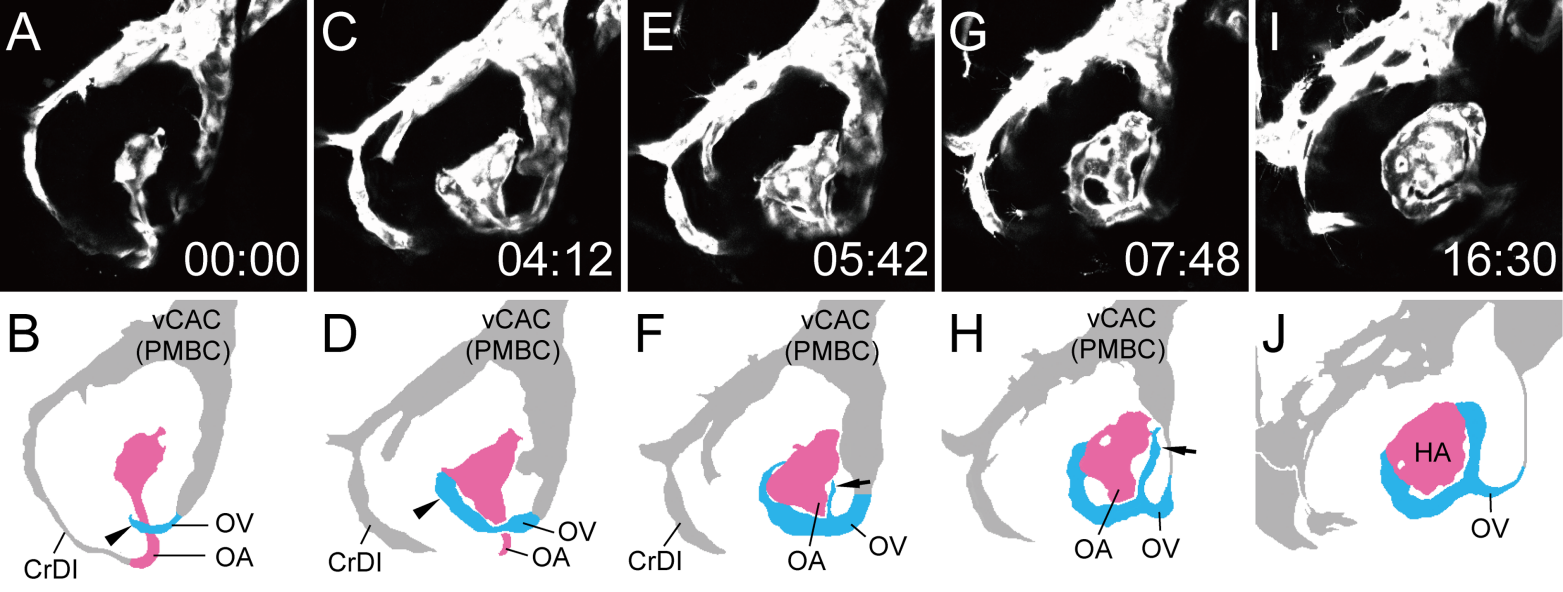
Circular vessel formation connecting the OA with the OV. Selected time-lapse images of a living *Tg(flk1*:*EGFP)*^*k7*^ embryo from 1 dpf (S4 Movie) (A, C, E, G and I) and their schematic diagrams (B, D, F, H and J). The time (hours:minutes) from the first frame is labeled in each image (A, C, E, G and I). Rostral is facing left and dorsal is facing upward. The formation of the left hyaloid vasculature was mainly observed. To visualize the formation of circular vessel connecting the OA with the OV, only the selected slices from S3 Movie were projected. Ocular vessels in the schematic diagrams are colored (OA: pink, and OV: sky blue). Arrowheads in B and D indicate the rostral sprout from the OV. Arrows in F and H indicate the caudal sprout from the OV.

In the lateral view, we further visualized the formation of the circular vessel anastomosing OA and OV (Fig 6). For this purpose, we extracted the selected slices from the S3 Movie and produced S4 Movie. The OV first connected with the OA at the rostral portion (Fig 6B and 6D arrowheads), and then the thin sprout from the OV extended caudally and connected with the caudal part of the OA to form the circular vessel beneath the lens (Fig 6F and 6H arrows).

### Integration of the hyaloid and ciliary vascular systems and the CVP formation

**Fig 7:**
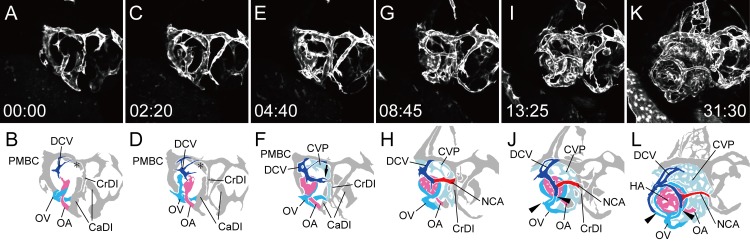
Integration of the hyaloid and ciliary vascular systems (rostral-lateral view). Selected time-lapse images of a living *Tg(flk1*:*EGFP)*^*k7*^ embryo from 1.25 dpf (S5 Movie) (A, C, E, G, I, and K) and their schematic diagrams (B, D, F, H, J, and L). The time (hours:minutes) from the first frame is labeled in each image (A, C, E, G, I, and K). Rostral is facing right and dorsal is facing upward. The formation of the right ocular vasculature, including the lateral transfer of the OV and the integration of the two systems via the IOC, were observed. Ocular vessels in the schematic diagrams are colored (NAC: red, DCV: blue, OA: pink, OV: sky blue, and CVP: light blue). Arrow in F indicates the connecting portion of the NCA in formation and the CrDI. Arrowheads in J and L indicate the forming IOC, which connects the hyaloid vascular system with the ciliary vascular system.

As observed above in both dorsal and lateral views of the time-lapse movies, the primary hyaloid and ciliary vascular systems were organized independently in early ontogeny. The former is distributed inside of the optic vesicle, while the latter is distributed to the surface of the eyeball. To complete the formation of the primary ocular vasculature, the OV transferred laterally to anastomose the two vascular systems. To visualize this process, we observed a *Tg(flk1*:*EGFP)*^*k7*^ embryo from 1.25 dpf in a rostral-lateral view (S5 Movie). By 1.25 dpf, the OA and OV had already anastomosed in the optic vesicle, and endothelial cell sprouting from the PMBC to form the DCV was observed (Fig 7A and 7B). After the primary integration of the OA and OV, the OV started to transfer laterally (Fig 7E–7I). The NCA from the DCV also sprouted and finally connected with the CrDI (Fig 7F arrow). The HA started to form the vascular plexus, and the rostral and caudal connection with the OV was formed (Fig 7G and 7H). After the lateral transfer of the OV, the hyaloid and ciliary vascular systems were integrated via the IOC on the lens. The rostral and caudal sprouts from the OV extended circularly on the edge of the lens and anastomosed with the NCA and DCV, respectively (Fig 7J and 7L arrowheads).

**Fig 8:**
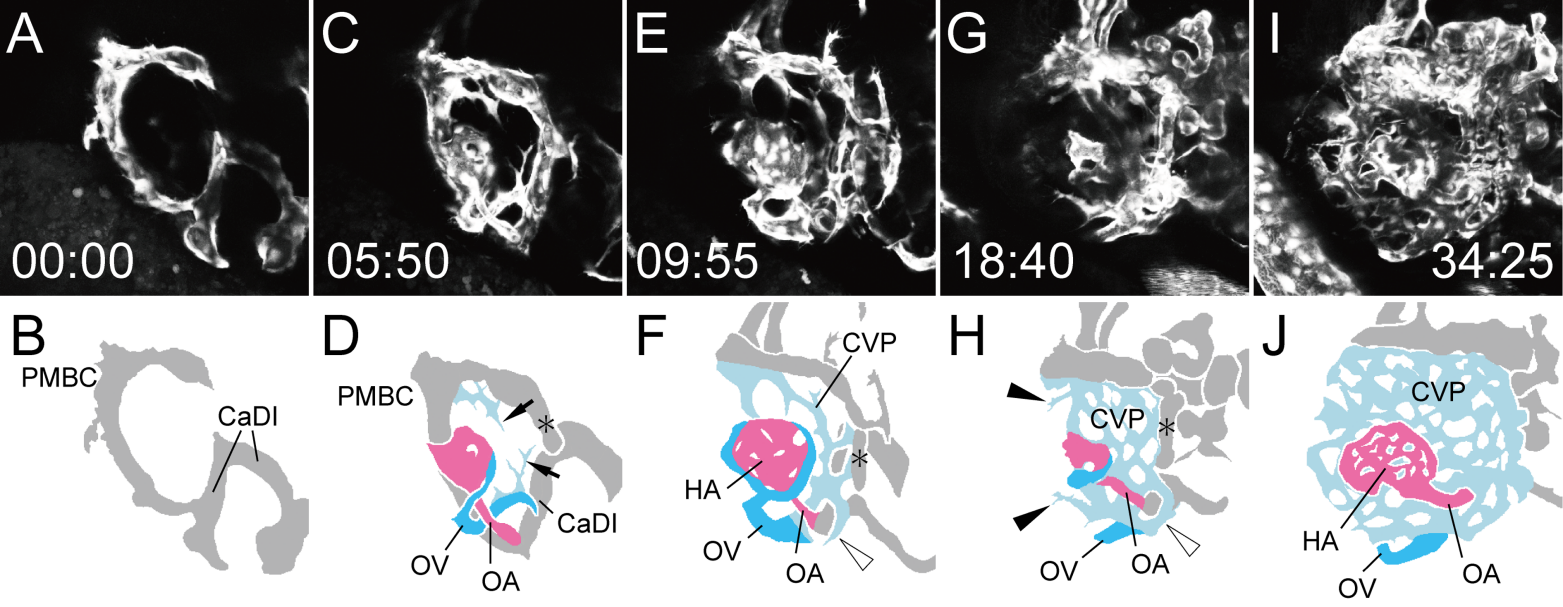
Formation of the CVP. Selected time-lapse images of a living *Tg(flk1*:*EGFP)*^*k7*^ embryo from 1.25 dpf (S6 Movie) (A, C, E, G, and I) and their schematic diagrams (B, D, F, H, and J). The time (hours:minutes) from the first frame is labeled in each image (A, C, E, G, and I). Rostral is facing right and dorsal is facing upward. To visualize CVP formation, only the selected slices from S5 Movie were projected. Ocular vessels in the schematic diagrams are colored (OA: pink, OV: sky blue, and CVP: light blue). Arrows in D indicate the rostral sprouts forming the CVP. Black arrowheads in H indicate the caudal sprouts forming the CVP. White arrowheads in F and H indicated the vascular plexus of the OV surrounding the OA. Asterisks in D, F and H indicates the new connection from the dorsal branch of the PMBC to the aCAC.

At the same time, the CVP (sky blue vessels) surrounding the eyeball was also organized (Fig 8). To visualize CVP formation, we again extracted the selected slices from S5 Movie and produced S6 Movie. The sprouts from the ventral branch of the PMBC and the root of the OV first connected with each other to form the vascular plexus in the rostral region of the eyeball (Fig 8D arrows). Then, other caudal sprouts from the ventral branch of the PMBC formed the vascular plexus in the caudal region (Fig 8H black arrowheads). The dorsal branch of the PMBC formed a new connection with the aCAC beneath the CrDI (Fig 8D, 8F and 8H asterisks). The CVP finally incorporated both of the dorsal branch of the PMBC and this branch, resulting in a connection between the DCV and the CVP. The vascular plexus of the OV surrounding the OA was also incorporated into the CVP, resulting in a connection between the OV and the CVP (Fig 8F and 8H white arrowheads).

**Fig 9:**
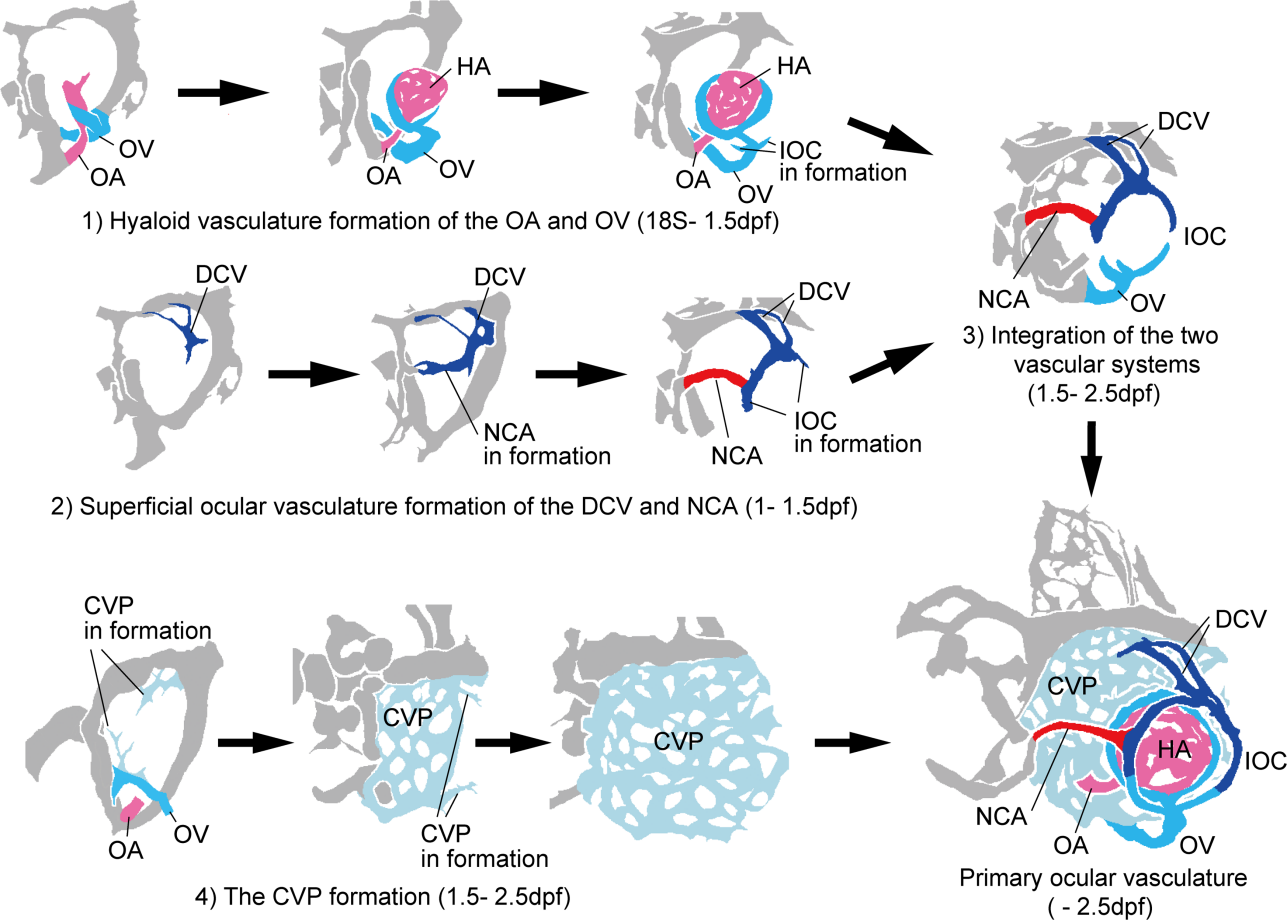
Schematic diagrams of primary ocular vasculature formation. Stepwise formation of the primary ocular vasculature, including the hyaloid vascular system of the OA and OV, the superficial ocular vascular system of the DCV and NCA, and the CVP, are indicated. Rostral is facing left and dorsal is facing upward. Ocular vessels in the schematic diagrams are colored (NAC: red, DCV: blue, OA: pink, OV: sky blue, and CVP: light blue).

In summary, the primary ocular vasculature formation consisted of four steps: 1) hyaloid vasculature formation of the OA and OV, 2) superficial ocular vasculature formation of the NCA and DCV, 3) lateral transfer of the OV to anastomose the two systems via the IOC, and 4) CVP formation around the eyeball. The stepwise formation of the ocular vasculature described above is summarized in Fig 9.

### Histological analysis of the lateral transfer of the optic vein

**Fig 10:**
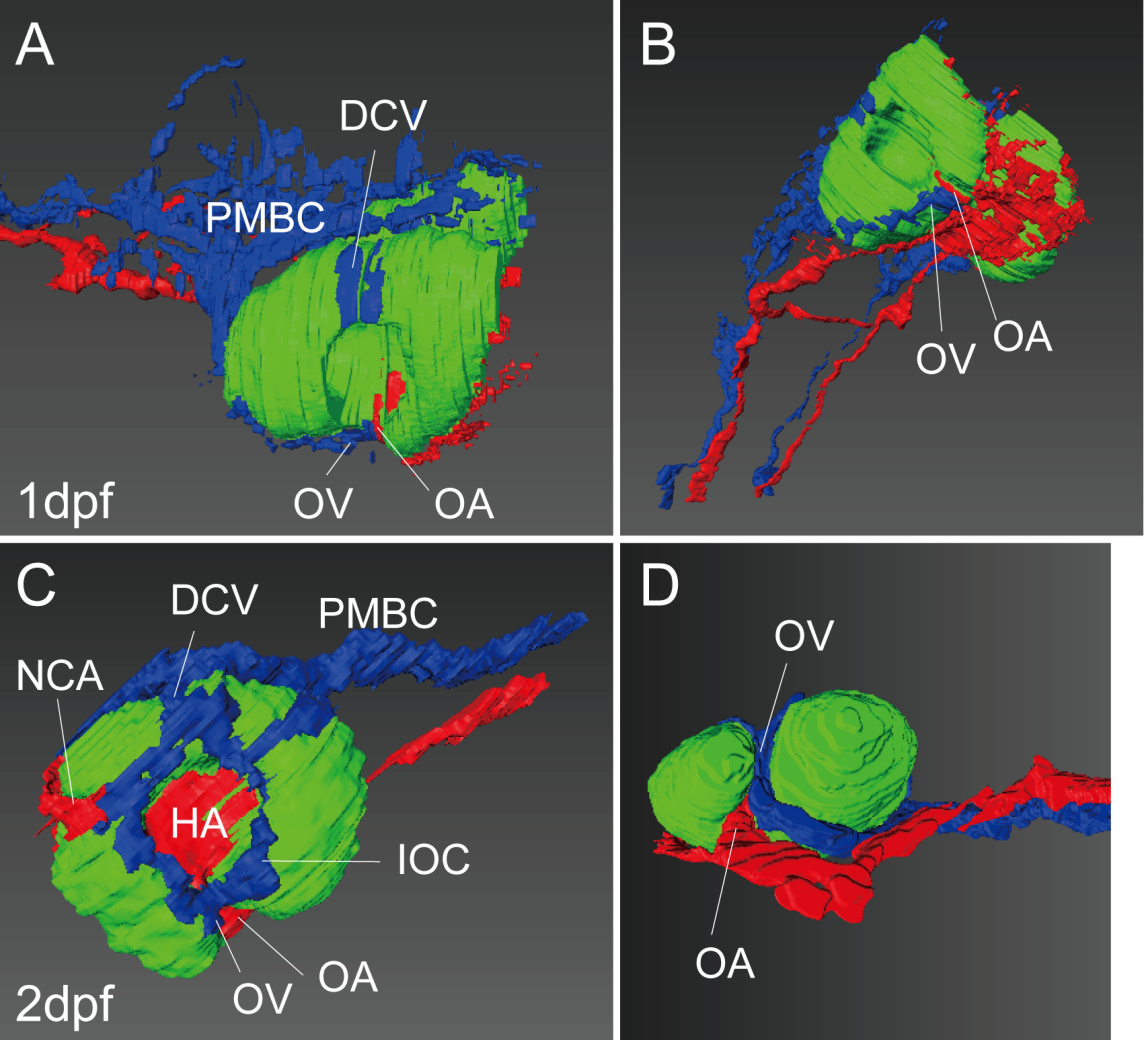
Three-dimensional reconstruction of the ocular region. Reconstructed images of the serial sections of GMA-embedded (A and B) and TECHNOVIT 7100-embedded (C and D) WT zebrafish embryos at 1(A and B) and 2(C and D) dpf. Lateral (A and C), ventral-lateral (B) and ventral (D) views. The artery, vein, and optic vesicle are colored in red, blue, and green, respectively.

Our time-lapse movie clearly demonstrated the lateral transfer of the OV that integrated the two ocular vasculatures. We analyzed this event histologically to determine how this transfer occurred (Fig 10); the relationship of the OA and OV with the surrounding tissue was analyzed. Serial sections of the GMA-embedded (1dpf) and TECHNOVIT 7100-embedded (2dpf) WT embryos were prepared and then three-dimensionally reconstructed with Amira (Fig 10). For visualization, we colored the artery, vein, and optic vesicle in red, blue, and green, respectively. As a result, we confirmed that the OA and OV entered into the optic vesicle via the optic fissure at 1 dpf (Fig 10A and 10B). As ontogeny progressed, the optic fissure was closing, and only the OA incorporated into the optic nerve. In contrast, the OV was observed at the outside of the closing portion (Fig 10C and 10D). Representative images of the serial sections are shown to demonstrate how to identify each vessel (S2 Fig). For the venous vessels, we first identified the PMBC at the caudal region of the optic vesicle. Then, we traced each venous vessel from the PMBC. For the arterial vessels, we first identified the PICA, and then traced each arterial vessel for the reconstruction.

## Discussion

In this study, we elucidated the precise anatomical architecture of the primary ocular vasculature in zebrafish (Fig 1) and its formation using time-lapse images of living *Tg(flk1*:*EGFP)*^*k7*^ embryos (Figs 3–9). We were the first to succeed in capturing the entire primary ocular vasculature formation event, in particular, the circular integration of the OA and OV, lateral transfer of the OV to integrate the hyaloidal and superficial ocular vasculatures, and formation of the CVP. We also elucidated the origin of each ocular vessel; the OA organized from the aCAC, the OV from the ventral-caudal part of the vCAC (PMBC), the DCV from the middle portion of the vCAC (PMBC), the NCA from the DCV, and the CVP from the vCAC (PMBC). These results indicate that almost all ocular vessels were organized from the vCAC (PMBC), with the exception of the OA. It is interesting that the arterial endothelial cells of the NCA were supplied from the venous vessel (DCV), as this type of arterial formation was also observed in the formation of the basilar artery and the central arteries [19].

As mentioned in the introduction, some researchers have studied the formation of the ocular vasculature in zebrafish. Isogai et al. (2001) first precisely described the vascular anatomy of a developing zebrafish by using confocal microangiography with fluorescent beads. They elucidated the entire vasculature of developing embryos at each stage, including the primary ocular vasculature consisting of the OA, OV, NCA, DCV, and CVP [11]. However, the microangiography approach could visualize only the lumenized vessels and not the endothelial cells; hence, the behavior of unlumenized endothelial cells forming the primary vasculature could not be analyzed. To overcome this methodological limitation, transgenic lines comprising endothelial cells that specifically expressed a fluorescent protein were established [15, 26]. The combination of these transgenic lines and multiphoton microscopy enabled us to perform time-lapse observations to analyze the primary vasculature formation including the ocular vasculature [15, 16, 18, 24]. The primary ocular vasculature in zebrafish was divided into the following two categories: the hyaloidal vasculature consisting of the OA and OV, and the ciliary vasculature consisting of both the superficial vasculature of NCA and DCV, and the CVP. Regarding the primary cranial vascular formation, using a *BAC Tg(etv2*:*EGFP)* zebrafish model Proulx et al. (2010) revealed that the two angioblast clusters of ROC and MOC establish almost all the cranial vasculature including the primary ocular vasculature. They also reported that while the ROC differentiated into the arterial angioblasts, the MOC differentiated into both the arterial (PICA) and venous (PMBC) angioblasts [18]. In this study, we named the arterial and venous angioblasts from the ROC as aCAC and vCAC, respectively, and live-imaged the primary ocular vasculature arising from them. For the ocular vasculature formation, Kitabi et al. (2009) demonstrated the early development of both the hyaloidal and ciliary vascular systems by imaging the *Tg(fli1*:*EGFP)* and *Tg(flk1*:*EGFP)* embryos at each stage from 18 hpf to 30 dpf [23]. Hartsock et al. (2014) also described the maturation of the hyaloidal vasculature and revealed the role of the lens in this process of maturation [22]. In this study, we describe the extension of OA and OV from the aCAC and vCAC (PMBC) respectively. For the first time, this work also revealed the stepwise integration of the OA and OV inside the optic vesicle, and the vascular plexus of the OV surrounding the OA, which contributed to the CVP formation. Regarding the superficial ocular vasculature formation, Kaufman et al. (2015) revealed the DCV and NCA formation from the PMBC, and the arterial differentiation of the NCA by the extension of the DCV via Notch activation. Additionally, using the Kaede tracing assay, they demonstrated that the OV derived from the PMBC connected with the superficial ocular vasculature [24]. We revealed the origin of the OV and the integration process of the hyaloidal and superficial ocular vasculatures by time-lapse analysis. Additionally, our time-lapse movie from 1.25 dpf (S5 and S6 Movies) captured not only the lateral transfer of the OV to integrate the two systems but also the CVP formation of the ciliary vascular system. Although, Isogai et al. (2001) described the existence of the CVP, they did not describe its formation in detail [11]. Our live imaging is the first report of CVP formation. We demonstrated the formation of hyaloidal and ciliary vasculatures and their integration in zebrafish. Although the previous studies elucidated only partial details of the formation of primary ocular vasculature, we have described these in detail in this study. The overview described herein will be valuable for precise understanding of the formation of primary ocular vasculature and for elucidation of the regulatory mechanisms.

To explore the OV transfer, we analyzed the optic vesicle of WT embryos at 1 and 2dpf histologically (Fig 10). The OA and OV entered into the optic vesicle via the optic fissure at 1dpf, and as ontogeny progressed, only the OV transferred laterally. The closure of the optic fissure was impaired in the lmo2 mutant owing to the abnormal size of the OV [29]. We speculated that the closure of the optic fissure occurred between the OA and OV, resulting in lateral transfer of the OV only, because the OV might be located outside of the closed portion (Fig 10D). The OA was included in the closing optic vesicle and consequently penetrated the optic nerve.

For decades, researchers have studied how the ocular vasculature forms in vertebrate embryos [6, 30]. In humans, two vessels, the primitive dorsal and ventral ophthalmic arteries, first distribute to the ocular region and supply blood to the vascular plexus surrounding the optic vesicle. These two arteries then form the hyaloid and ciliary arteries. In the following remodeling process, the primitive dorsal and ventral ophthalmic arteries regress, and instead the ophthalmic artery from the internal carotid artery supplies blood to the retina via the central retinal and ciliary arteries [6]. The stapedial artery also temporarily distributes to the orbit and regresses as ontogeny progresses in human embryos. In the mouse embryo, the two inner ophthalmic arteries from the internal carotid artery are first organized. As ontogeny progresses, these arteries regress, and instead the outer ophthalmic artery from the stapedial artery supplies blood to the ocular region [30]. These two inner ophthalmic arteries in mouse embryos may correspond to the primitive dorsal ophthalmic artery in human embryos. In the mouse embryo, the vascular plexus surrounding the optic vesicle also formed. From the ventral portion of the annular vessel on the lens, the vascular sprout entered into the optic vesicle and formed the intraocular vessel. Finally, this vessel connected with one of the inner ophthalmic arteries and differentiated into the HA [30]. In zebrafish, the hyaloidal vasculature formed first and then the superficial ocular vasculature and the CVP formed. In contrast, the vascular plexus, which may correspond to the superficial ocular vasculature and the CVP of the zebrafish, was formed first in the human and mouse embryos. The ophthalmic artery which entered into the optic vesicle and connected with the HA formed a short while later. The primary ocular vasculature, consisting of the hyaloidal and superficial ocular vasculatures and the CVP, indicated similarity between the vertebrate phyla, though the formation process was different between them. While zebrafish embryos usually hatch at 2 dpf and start eating at 5 dpf, the retinal vasculature in the mouse is formed after birth. Thus, the requirement of visual function may be involved in the ocular vasculature formation and understanding the regulatory mechanisms involved will be necessary to resolve this question.

Recently CRISPR/Cas9 has emerged as a convenient and efficient method to edit targeted genome sequences [31–34], and some genes responsible for genetic diseases associated with abnormal ocular vasculature already have been elucidated [35–41]. In particular, the defect of the developing ocular vasculature was demonstrated in the *ZNF408* deficient embryo of zebrafish [35]. We also try to establish the knockout line of the some genes which may be associate with the ocular vasculature formation using this system. Furthermore we succeeded in regulating spatio-temporal gene expression in targeted single cells using infrared laser, and this method will be useful for rescue of the deficient gene in the targeted cell [42]. By combining these new methods with the morphological and genetic information, we will elucidate both the morphogenetic and pathogenic mechanisms of ocular vasculature formation in future.

## Conclusions

In this study, we demonstrated the precise anatomical architecture of the primary ocular vascular formation in zebrafish and entire process of the formation using the living *Tg(flk1*:*EGFP)*^*k7*^ zebrafish embryos. The primary ocular vascular formation consisted of four steps, hyaloid vasculature formation, superficial ocular vasculature formation, lateral transfer of the OV to anastomose the two vascular systems, and CVP formation. We precisely described the stepwise procedures of the formation. To overcome visual dysfunction induced by congenital and acquired diseases of the ocular vasculature, we need to understand the regulatory mechanisms underlying this process. We believe the morphological information on ocular vasculature formation presented here will be useful for this purpose.

## Supporting information

S1 Fig Arterial and venous characteristics of each ocular vessel. Light-sheet microscopy of the double *Tg(flt1_enh*:*EGFP)*^*k28*^ and *Tg(flk1*:*mCherry)*^*k6*^ zebrafish embryos at 18S (A-C), 24S (D-F), and 1.5 dpf (G-I). *Tg(flt1_enh*:*EGFP)*^*k28*^ (A, D, and G), *Tg(flk1*:*mCherry)*^*k6*^ (B, E, and H), and merged (C, F, and I) images. Dorsal (A-C), rostral-lateral (D-F), and lateral (G-I) views. Only the arterial components of the ocular vasculature, aCAC, OA, and NCA, expressed EGFP, whereas all vessels expressed mCherry. Asterisk in D indicate the PMBC which did not express EGFP.
(TIF)

S2 Fig Selected images of serial sections before the reconstruction. Selected images of the serial sections at 1 (A-C) and 2 (D-G) dpf. Frontal (A-C) and horizontal (D-G) planes. Each ocular vessel and the positioning number of each selected image were indicated.
(TIF)

S1 Movie Time-lapse movie of the primary ocular vasculature formation (dorsal view). Time-lapse imaging of the living *Tg(flk1*:*EGFP)*^*k7*^ embryo from 18S for 27 h in a dorsal view. Z stacks were obtained at 18-min intervals. Rostral is downward and the right side faces left. The formation of the left ocular vasculature was mainly observed. Movie frame rate: 6/s.
(AVI)

S2 Movie Time-lapse movie of the OV extension surrounding the OA. Time-lapse imaging of a living *Tg(flk1*:*EGFP)*^*k7*^ embryo from 18S for 15 h in a dorsal view. Z stacks were obtained at 18-min intervals. Rostral is downward and the left side faces right. To visualize the formation of the ventral vasculature, only ventral slices were extracted from S1 Movie. The formation of the left ocular vasculature in the ventral region was mainly observed. Movie frame rate: 6/s.
(AVI)

S3 Movie Time-lapse movie of the primary ocular vasculature formation (lateral view). Time-lapse imaging of a living *Tg(flk1*:*EGFP)*^*k7*^ embryo from 15S for 27 h in a lateral view. Z stacks were obtained at 18-min intervals. Rostral is facing left and dorsal is facing upward. The formation of the left ocular vasculature was mainly observed. Movie frame rate: 6/s.
(AVI)

S4 Movie Time-lapse movie of the circular vessel formation connecting the OA with the OV (lateral view). Time-lapse imaging of a living *Tg(flk1*:*EGFP)*^*k7*^ embryo from 1 dpf for 18 h in a lateral view. Z stacks were obtained at 18-min intervals. Rostral is facing left and dorsal is facing upward. To visualize the formation of the inner circular vessel of the hyaloid vascular system, selected slices were extracted from S3 Movie. Movie frame rate: 6/s.
(AVI)

S5 Movie Time-lapse movie of the integration of the hyaloid and ciliary vascular systems (rostral-lateral view). Time-lapse imaging of a living *Tg(flk1*:*EGFP)*^*k7*^ embryo from 1.25 dpf for 34 h in a rostral-lateral view. Z stacks were obtained at 35-min intervals. Rostral is facing right and dorsal is facing upward. The formation of the right ocular vasculature, including the lateral transfer of the OV and CVP formation were observed. Movie frame rate: 4/s.
(AVI)

S6 Movie Time-lapse movie of the CVP formation (rostral-lateral view). Time-lapse imaging of a living *Tg(flk1*:*EGFP)*^*k7*^ embryo from 1.25 dpf for 34 h in a rostral-lateral view. Z stacks were obtained at 35-min intervals. Rostral is facing right and dorsal is facing upward. To visualize CVP formation, selected slices were extracted from S5 Movie. Movie frame rate: 4/s.
(AVI)

## Abbreviations

aCAC: arterial cerebral angioblast cluster
ACeV: anterior cerebral vein
CaDI: caudal division
CrDI: cranial division
CVP: choroidal vascular plexus
DCV: dorsal ciliary vein
HA: hyaloid artery
IOC: inner optic circle
LDA: lateral dorsal aorta
MOC: midbrain organizing center
NCA: nasal ciliary artery
OA: optic artery
OV: optic vein
PICA: primitive internal carotid artery
PMBC: primordial midbrain channel
ROC: rostral organizing center
vCAC: venous cerebral angioblast cluster
