# Supplementary figures and images for: Live imaging of primary ocular vasculature formation in zebrafish
Source: PLoS One. 2017 Apr 26;12(4):e0176456. doi: 10.1371/journal.pone.0176456 (PMC5405983; doi:10.1371/journal.pone.0176456)

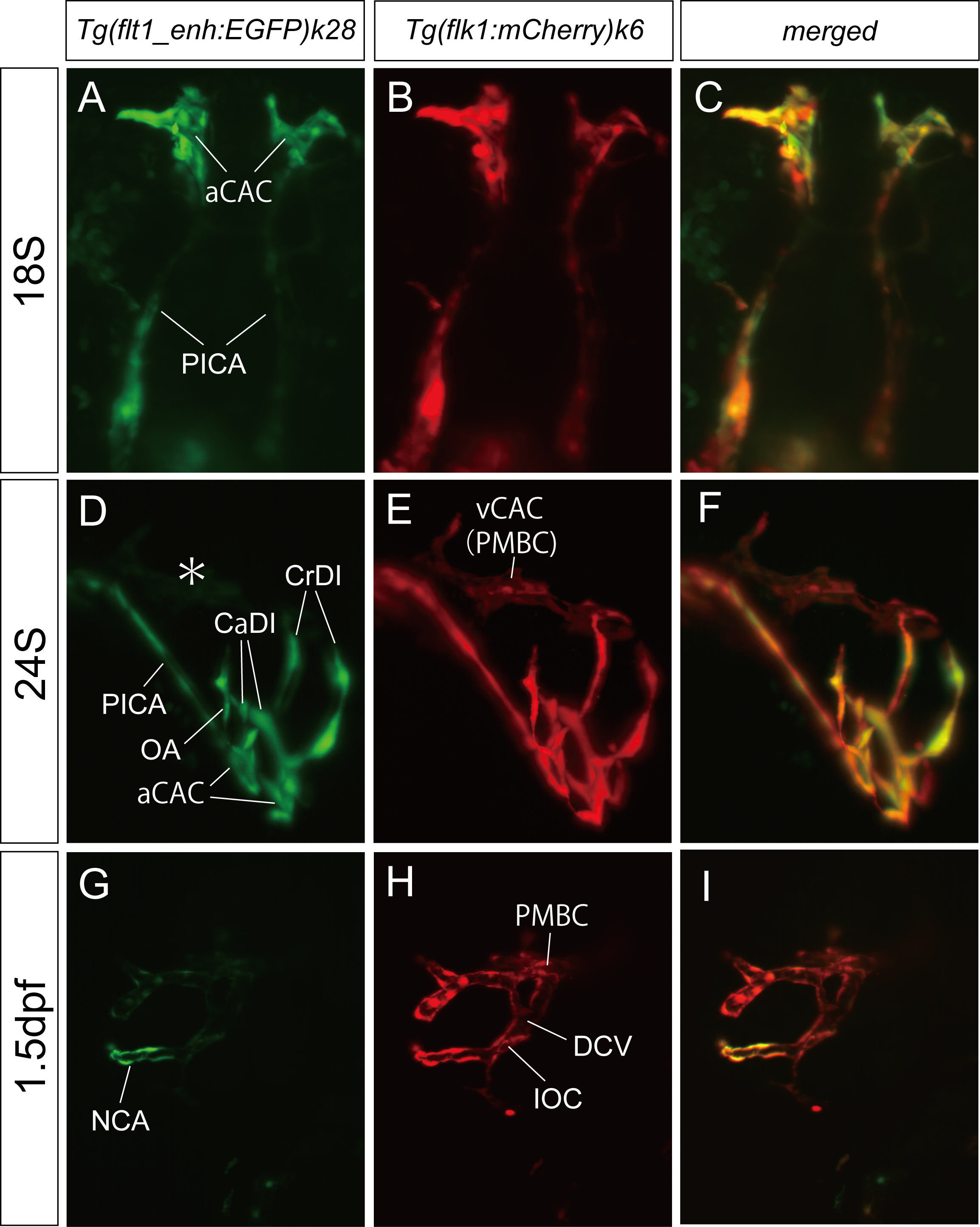

Supplement: S1 Fig — Light-sheet microscopy of the double Tg(flt1_enh:EGFP)k28 and Tg(flk1:mCherry)k6 zebrafish embryos at 18S (A-C), 24S (D-F), and 1.5 dpf (G-I). Tg(flt1_enh:EGFP)k28 (A, D, and G), Tg(flk1:mCherry)k6 (B, E, and H), and merged (C, F, and I) images. Dorsal (A-C), rostral-lateral (D-F), and lateral (G-I) views. Only the arterial components of the ocular vasculature, aCAC, OA, and NCA, expressed EGFP, whereas all vessels expressed mCherry. Asterisk in D indicate the PMBC which did not express EGFP. (TIF) [file pone.0176456.s001.tif]

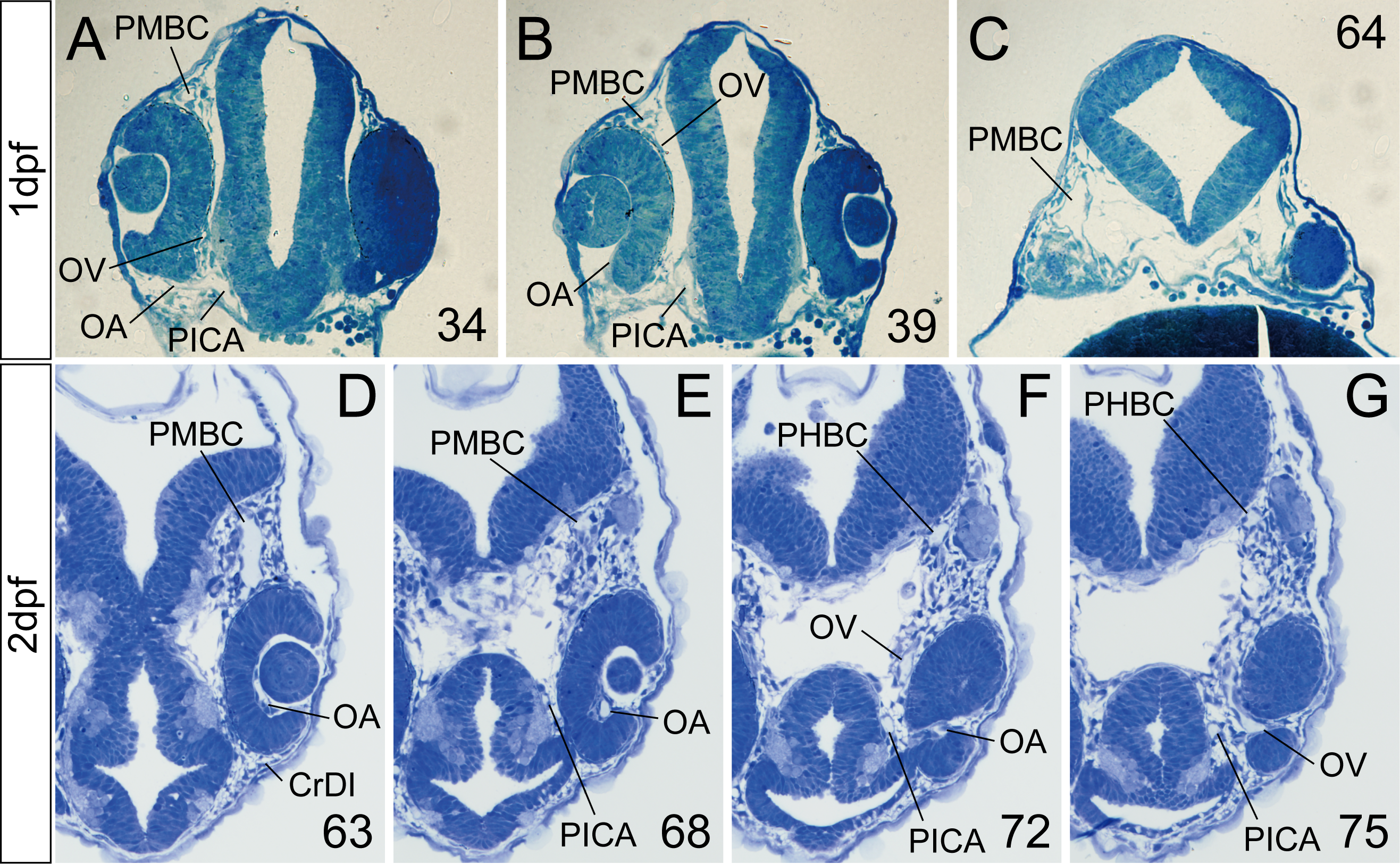

Supplement: S2 Fig — Selected images of the serial sections at 1 (A-C) and 2 (D-G) dpf. Frontal (A-C) and horizontal (D-G) planes. Each ocular vessel and the positioning number of each selected image were indicated. (TIF) [file pone.0176456.s002.tif]
